# The Impact of Gender on the Effectiveness of an Auricular Acupressure Intervention Administered to Community-Dwelling Poor Sleepers: A Cluster Randomized Controlled Trial

**DOI:** 10.1097/JNR.0000000000000427

**Published:** 2021-03-19

**Authors:** Hsuan-Man HUNG, Hsiao-Ching CHIANG, Hui-Ling WANG

**Affiliations:** 1 PhD, RN, Associate Professor, Department of Nursing, Fooyin University, Kaohsiung City, Taiwan, ROC; 2 MSN, RN, Lecturer, Department of Nursing, Tajen University, Yanpu City, Taiwan, ROC; 3 PhD, RN, Assistant Professor, Department of Nursing, Fooyin University, Kaohsiung City, Taiwan, ROC.

**Keywords:** auricular acupressure, sleep quality, gender effects

## Abstract

**Background:**

Women report a higher incidence of sleep problems than men. Few studies addressing the effect of gender on the efficacy of administering auricular acupressure (AA) at shenmen points (heart meridian 7 [HT7]) on sleep quality have been published.

**Purpose:**

The primary aim of this study was to investigate the effects of a 4-week AA intervention applied at the HT7 points on sleep quality, perceived physical health, and perceived mental health in community-dwelling individuals with poor self-reported sleep quality. Additional analyses were used to evaluate the gender-specific effects of this intervention.

**Methods:**

A cluster randomized controlled trial with repeated-measures design was used. One hundred seventy-nine eligible participants were randomly assigned to either the AA group (*n* = 88; 47 women, 41 men) or the sleep hygiene instruction (SHI) group (*n* = 91; 52 women, 39 men). The AA group self-administered acupressure at HT7 on both ears for a 4-week period, whereas the SHI group received an SHI information sheet. Outcome measures included the Pittsburgh Sleep Quality Index (PSQI) and the Short-Form Health Survey-12 Version 2, with data collected at baseline and at 2, 4, and 8 weeks posttest.

**Results:**

Linear mixed-model analysis revealed that the participants in the AA group experienced significantly greater reductions in mean PSQI global score and the three indices of sleep latency, subjective sleep quality, and daytime dysfunction than the SHI group at 2 and 4 weeks posttest. The improvements in subjective sleep quality and daytime dysfunction remained at 4 weeks posttest in the AA group, but not in the SHI group. The PSQI global score decreased significantly more in men than women in the AA group between baseline and 4 weeks posttest.

**Conclusions/Implications for Practice:**

Four weeks of self-administered acupressure at HT7 on both ears is an effective intervention for community-dwelling poor sleepers who are over 45 years old. Moreover, the improvements in subjective sleep quality and daytime dysfunction persist for up to 4 weeks after the end of the intervention. This self-administered acupressure intervention is more effective in men than in women in terms of improving sleep quality. Gender bias is known to influence research results and may lead to inappropriate generalizations. Thus, future studies that are performed to build basic scientific evidence should include considerations of the effects of gender in the study design.

## Introduction

Sleep disturbance, which is known to adversely affect physical and psychological health outcomes, currently affects 31%–60% of adults aged 45 years and older and is a health problem that is increasing sharply among postmenopausal women (Jehan et al., 2015; Kerkhof, 2017; Manjavong et al., 2016). Gender should be considered when diagnosing and treating sleep disturbance, as women are more likely than men to report longer sleep latency, shorter sleep duration, and breathing-related sleep disorders (Auer et al., 2018; Jehan et al., 2015; Mallampalli & Carter, 2014). The side effects of sedative drugs often lead poor sleepers to seek nonpharmacological interventions, of which acupressure has been shown to be highly effective (Huang et al., 2018). Acupressure is a traditional Chinese medicine (TCM) technique. Rather than using acupuncture needles, auricular acupressure (AA) applies thumb or finger pressure, usually together with seeds or magnetic stones, to specific acupoints for therapeutic effect (Cheng et al., 2015; Vagharseyyedin et al., 2019). AA does not require sophisticated equipment or invasive procedures and may be administered by physicians and nurses or even self-administered, which may increase its acceptability to patients (Arai et al., 2013; Cheng et al., 2015).

According to the theoretical foundations of TCM, the ears are a microcosm of the human body and connect directly or indirectly to the 12 meridians, the stimulation of which facilitates the internal balance of Qi (energy) flow, which is necessary to maintain and improve health (Cheng et al., 2015; Song et al., 2015). The effectiveness of AA in improving sleep quality and reducing stress, pain, fatigue, anxiety, burnout, depressed moods, and systolic blood pressure has been demonstrated in quasi-experimental, randomized controlled trial, systematic review, and meta-analysis of studies on subjects including middle-aged and older women (Cha et al., 2017; Kao et al., 2012; Lo et al., 2013), women who have given birth by cesarean section (Kuo et al., 2016), nursing students (Chueh et al., 2018), healthcare employees (Olshan-Perlmutter et al., 2019), institutionalized residents (Chen et al., 1999; Reza et al., 2010), and patients undergoing treatment for hypertension (Gao et al., 2020), leukemia (X. R. Liu et al., 2020), neck pain (Lee & Park, 2019), chemoradiotherapy (Chou et al., 2019), hemodialysis (Tsay & Chen, 2003; Wu et al., 2018), and low back pain (Yang et al., 2017). Some of the previous studies applied AA in combination with acupoint massage (e.g., head, hands, and feet), with the shenmen (heart meridian 7 [HT7]) acupoints on the ears and hands most frequently targeted to improve sleep quality (Cha et al., 2017; Reza et al., 2010; Wu et al., 2018). The results of a systematic review and meta-analysis of studies exploring the use of acupressure to promote sleep quality revealed that the Pittsburgh Sleep Quality Index (PSQI) is the most commonly used outcome measure and 1–8 weeks is the most commonly studied interval, with acupressure sessions held 3–5 times per day for periods of between 3 weeks and 12 months (the most common duration was 4 weeks), pressure maintained at each point for 2–5 minutes, and the *Deqi* sensation (i.e., soreness, warm, numbness, or distention) identified as the primary indicator of optimal pressure (Hmwe et al., 2019; Waits et al., 2018). Moreover, applying less pressure to the ear acupoints than the hand acupoints (250–300 g vs. 3–5 kg; Cheng et al., 2015; Kuo et al., 2016; Tsay & Chen, 2003) and AA to the HT7 points is simple to achieve and may be suitable for self-administration among middle-aged and older adults (Lo et al., 2013; Hmwe et al., 2019). Tsou (2018) reported different sleep problems and different attitudes toward related treatments among older women and men. In addition, Lund and Lundeberg (2008) reported that gender influences therapist–patient interactions and highlighted the importance of gender when assessing the patient response to interventions.

In the chronic care model proposed by Wagner et al. (1996), patients play a critical role in managing their health using community resources, with healthcare providers encouraged to empower patients to establish management techniques (Grover & Joshi, 2014). However, the early identification of sleep disturbance and the timely implementation of interventions to improve gender-specific sleep problems are crucial for self-management support. Sleep hygiene is an intervention that is commonly implemented in clinical practice (Maness & Khan, 2015). Few studies have studied the effects of AA on sleep hygiene, or how sleep quality differs among men and women after applying AA at the HT7 points without the coordination of acupoints. Sleep disturbance has been associated with poor self-perceived physical and mental health (Y. Liu et al., 2013), although results that contradict this association have been reported in Kao et al. (2012) and Cheung et al. (2020). Thus, the primary aim of this study was to investigate the effects of a 4-week AA intervention applied at the HT7 points on sleep quality, perceived physical health, and perceived mental health in community-dwelling individuals with poor self-reported sleep quality. Additional analyses were used to evaluate the gender-specific effects of this intervention.

## Methods

### Study Design and Setting

A cluster randomized controlled trial was conducted with a purposive sample of participants from a community in southern Taiwan. Two lots, one marked A for the AA group and the other marked S for the sleep hygiene instruction (SHI) group, were applied to conduct permuted block randomization. A unit was defined as either a community activity center or a community care center. The participants for this study were recruited from 12 community activity centers or community care centers located in two nearby cities that had not instituted a TCM program during the previous year and that did not have a TCM program planned for the next 8 weeks. Each participating community was assigned randomly to either the AA group (receiving an AA [six communities]) or the SHI group (receiving sleep information in the form of a printed sheet of SHI [six communities]). The risk of intergroup contamination was reduced by using cluster randomization. Each center was managed by the same intervention administrator and assessment team for the duration of the study to promote compliance with the study protocol by participants and to help verify the data. Two research assistants were trained by the first author to ensure interrater consistency in data collection and were blinded to the procedure that was used to collect data. The institutional review board of the Antai Medical Care Corporation Antai Tian-Sheng Memorial Hospital approved the study protocol (IRB Approval No. 16-068-B1), and all of the participants provided written informed consent. This study was implemented between August 2016 and May 2017.

### Participants

**Figure 1.**
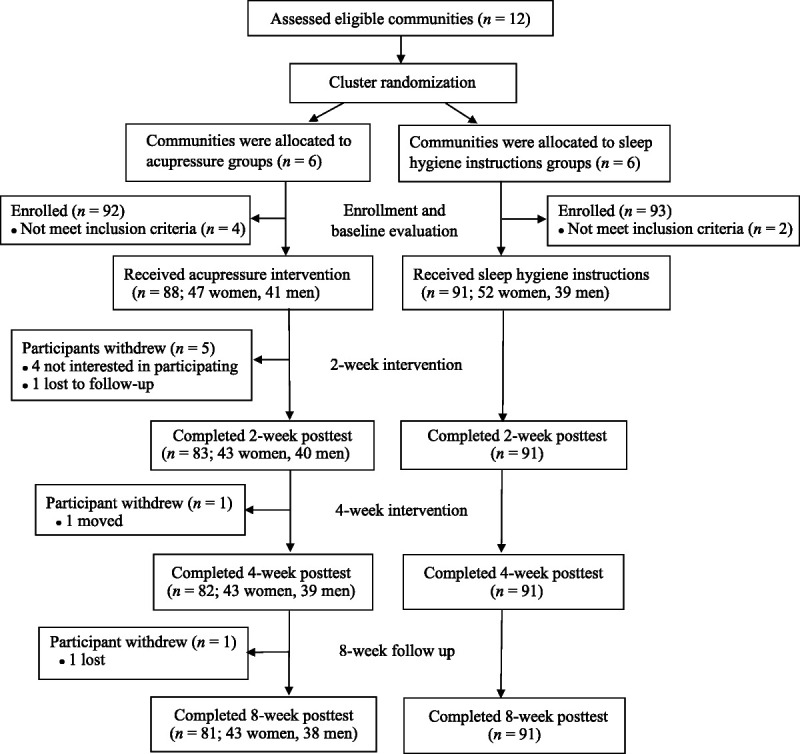
Flowchart of Participants

All of the potential participants were recruited from communities that are located in two cities in southern Taiwan. Inclusion criteria were (a) 45 years of age or older; (b) able to communicate in Mandarin or Taiwanese (interview or self-administration); (c) poor sleeper with a PSQI score of more than 5; (d) normal mental health status with no diagnosis of dementia or psychiatric disorders; and (e) no skin lesions, wounds, or dermatitis on either ears (participants in AA group only). Exclusion criteria were (a) having a current, severe medical condition (e.g., stroke, congestive heart failure, myocardial infarction, chronic obstructive pulmonary disease, or cancer) that interferes with nocturnal sleep; (b) currently receiving or having received in the previous 6-month period medical treatment or TCM for sleep problems; (c) currently pregnant; and (d) currently using hypnotic drugs more than once per week. All eligible individuals who expressed a desire to take part in this study were permitted to enroll. Thus, a total of 179 eligible individuals were enrolled as participants and assigned via cluster randomization to either the AA group (*n* = 88; 47 women, 41 men) or the SHI group (*n* = 91; 52 women, 39 men; Figure 1).

### Intervention

The protocol for the AA intervention was developed based on the literature (Cheng et al., 2015; Chueh et al., 2018; Hmwe et al., 2019; Tsay & Chen, 2003; Waits et al., 2018) and on consultations with a Chinese medicine (CM) physician who is an expert in acupuncture practice and has 20 years of experience in CM clinical practice and education. Two CM specialist qualified nurses selected the acupoint sites and intervention protocol by mutual consensus and conducted the AA intervention. The ear HT7 point is located deep in the triangular fossa of the auricle, inferior to the superior crus. Participants in the AA group applied AA on each ear at the HT7 point (starting with the left ear). After sterilizing the site with a 75% alcohol swab, the two nurse researchers taped a semen vaccaria seed on the acupoint and instructed participants to perform self-administered acupressure for a duration of 2 minutes, 4 times per day for 4 weeks. Each 2-minute application was implemented in a repeating cycle of 8 seconds of pressure, followed by 2 seconds of rest. The seed was removed after remaining in place for 5 full days. In the event that one or more seeds became dislodged prior to the end of the 5-day period, the researchers replaced the seed(s) as soon as possible and concurrently confirmed the participant’s compliance with the protocol and accurate self-performance of the intervention. In addition, written instructions on the AA protocol were provided to the participants in the AA group, and each participant was asked to record in an acupressure diary the frequency, time, and duration of each acupressure session as well as sensations (i.e., warmth, numbness, and soreness) felt during these sessions. The intervention group participants received AA either in the community activity/care center or at home.

The participants in the SHI group received a sleep hygiene information sheet that was developed based on a comprehensive review of the literature (Chu & Lu, 2011; Hung & Chen, 2011; Irish et al., 2015; Maness & Khan, 2015) conducted by researchers with doctoral degrees in nursing. Furthermore, the two other researchers involved in this study educated the participants individually using this sheet and were trained for consistent procedures of education and consultations by the first author. Face-to-face or telephone consultations were conducted multiple times with each participant over the 4-week period. The average frequency of consultation (face-to-face or telephone) for the SHI group was 1.13 times during the 4-week period.

### Measures

The demographic datasheet was completed by all of the participants at baseline, whereas the PSQI and the Short-Form Health Survey-12 Version 2 (SF-12v2) were completed by all of the participants at baseline and at 2, 4, and 8 weeks posttest.

#### Primary outcome measure

Sleep quality was measured using the PSQI (Buysse et al., 1989), which consists of 19 items in seven components, including subjective sleep quality, sleep latency, sleep duration, habitual sleep efficiency, sleep disturbances, use of sleeping medication, and daytime dysfunction. Individual items are scored on a 0–3 scale, and the seven component scores are summed to obtain the global PSQI score, which has a total possible score range of 0–21. A global score greater than 5 indicates a poor sleeper, with the PSQI providing a diagnostic sensitivity of 98.7% and a specificity of 84.4% for diagnosing sleep disturbances (Backhaus et al., 2002). The Chinese version of the PSQI has shown acceptable test–retest reliability over a 14- to 21-day interval, with a coefficient of .85, and is thus a valid instrument for assessing primary insomnia in community-based studies (Tsai et al., 2005). The Cronbach’s alpha was .74 in this study.

#### Secondary outcome measure

Perceived physical and mental health status was measured in this study using the SF-12v2, which is a shorter version of the 36-item Short Form Health Survey (SF-36) that was developed by Ware et al. in 2002. The SF-12v2 contains 12 items in eight subscales (physical functioning, role limitations due to physical problem, bodily pain, general health, vitality, social functioning, role limitations due to emotional problems, and mental health) and generates two summary scores (Physical Component Summary-12 [PCS-12] and Mental Component Summary-12 [MCS-12]). The PCS-12 and MCS-12 scores were calculated using the Quality Metric Health Outcomes Scoring Software 2. This software uses all of the 12 items to produce scores for the PCS-12 by combining and normalizing the role limitations due to physical problem, general health, bodily pain, and physical functioning scales and for the MCS-12 by combining and normalizing the role emotional, mental health, social functioning, and vitality scales. The total possible score for each component ranges from 0 to 100, with higher scores indicating better perceived physical or mental health status. The SF-12v2 has been evaluated and shown to provide good validity, reliability, and sensitivity in ethnically Chinese populations (Lam et al., 2013). The Cronbach’s alpha of the PCS-12 and the MCS-12 in this study was .80 and .76, respectively.

### Statistical Analysis

Analyses were conducted using IBM SPSS Statistics Version 22.0 (IBM, Inc., Armonk, NY, USA). The tests conducted for intergroup differences in terms of baseline characteristics and pretest variables were chi-square for categorical variables and two-sample *t* tests for continuous variables. Noncompliance and missing outcomes are two major complications encountered in longitudinal studies, especially randomized controlled trials (Son et al., 2012). Applying intent-to-treat (ITT) analysis, which uses an unbiased approach to assess treatment efficacy, is a potential solution to this problem. The data from all participants in both groups, regardless of adherence or withdrawal status, are considered in the ITT analysis (Gupta, 2011; Son et al., 2012). Each outcome measure was analyzed using a linear mixed model of covariates in the recommended set of longitudinal data to confirm that neither the statistical approach nor missing data corrupted the results (S. Liu et al., 2012). Treatment differences (fixed effects) were investigated using a mixed model, with both fixed and random items, whereas the correlated effects (random effects, including the clustering effect) were adjusted using repeated measures. The linear mixed model is able to accommodate missing data without multiple imputations, providing a natural way to deal with incomplete data (S. Liu et al., 2012; Son et al., 2012). The Bonferroni adjustment was used for multiple pairwise comparisons. The significance level for all of the analyses was set at *p* < .05.

In this study, sample size was calculated based on the primary outcome of between-group differences. As in Lo et al. (2013), in this study, the mean difference between groups was 4.16, the pool *SD* was 7.1, and the effect size was 0.58. A medium effect of 0.6, with a minimum sample of 45 per group, was required to achieve a power of 0.80, with an alpha of .05 (Plichta & Kelvin, 2013). However, the effects of gender were also considered in each group. Thus, 47 women and 41 men in the AA group and 52 women and 39 men in the SHI group were included in the study and in the ITT analyses.

## Results

Of the 179 initial participants, 172 completed the trial, including 81 (43 women and 38 men) in the AA group and 91 (52 women and 39 men) in the SHI group. The attrition rate was 8.0% in the AA group (four individuals were not interested in participating, two were lost to contact, and one moved away) and zero in the control group. None of the participants was excluded from the analysis due to the exclusion criterion of increased hypnotics use or receiving medical treatments for sleep problems after enrollment. The mean age of the participants was 66.2 years (59.6 years for the women and 73.61 years for the men) in the AA group and 62.8 years (60.9 years for the women and 65.3 years for the men) in the SHI group. The majority of participants were married (75.4%), living with their family (87.2%), and possessed either a junior high school or lower level of formal education (88.8%). More than half of the participants were unemployed (*n* = 116, 64.8%), were nonregular exercisers (*n* = 109, 60.9%), and had chronic diseases (*n* = 105, 58.7%). The three most prevalent sleep problems, in descending order, were prolonged sleep latency (mean score = 2.44), short sleep duration (mean score = 2.16), and poor subjective sleep quality (mean score = 2.10). As shown in Table 1, no significant intergroup differences were found in terms of demographics. Significant intergender differences were identified in the AA group in terms of mean age (*p* < .001) and level of education (*p =* .002).

**Table 1.** Comparison of Demographic Characteristics Between Groups (*N* = 179)

Characteristic	Acupressure Group (*n* = 88)		SHI Group (*n* = 91)		*t*/χ^2^	*p*
	*n*	%	*n*	%		
Gender					0.25	.615
Female	47	53.4	52	57.1		
Male	41	46.6	39	42.9		
Age (years), *M* and *SD*	66.2	12.0	62.8	12.3	1.85	.066
Education					5.47	.065
≤ Junior high school	41	46.6	58	63.7		
Senior high school	36	40.9	24	26.4		
≥ College	11	12.5	9	9.9		
Marital status					0.93	.629
Married	66	75.0	69	75.8		
Widowed	16	18.2	13	14.3		
Single/divorced	6	6.8	9	9.9		
Living with ^a^						.828
Family members	78	88.6	78	85.7		
Alone	9	10.2	12	13.2		
Others	1	1.2	1	1.1		
Employment status					0.00	.993
Yes	31	35.2	32	35.2		
No	57	64.8	59	64.8		
Chronic diseases					0.18	.675
Yes	53	60.2	52	57.1		
No	35	39.8	39	42.9		
Regular exercise					2.93	.087
Yes	40	45.5	30	33.0		
No	48	54.5	61	67.0		
Smoking status					0.05	.822
Yes	6	6.8	7	7.7		
No	82	93.2	84	92.3		
Alcohol consumption					0.03	.855
Yes	8	9.1	9	9.9		
No	80	90.9	82	90.1		

*Note.* SHI = sleep hygiene instruction.

^a^Fisher’s exact test.

Comparisons of the means and standard deviations for the outcome measures at baseline are shown in Table 2. Significant differences between the two indices of habitual sleep efficiency (*p* = .024) and sleep disturbances (*p* = .004) were found between the groups. Categorical variables were measured dichotomously, and the variables were dummy-coded before being entered into the mixed model (*yes* = 1, *no* = 0). Based on the results of analysis by gender, the pretest PSQI global (*p* = .049), sleep disturbance (*p* = .001), and sleep duration (*p* = .017) scores were significantly lower in women than in men in the AA group. Furthermore, the mixed-model analysis was used to adjust for significant variables between groups at baseline, the short- and long-term effects of the 4-week AA and SHI interventions on sleep quality, and perceived physical and mental health status. Results are presented in Table 3. Significant differences in the mean changes in the PSQI global score (*p* = .002) and in the three indices of sleep latency (*p* = .042), subjective sleep quality (*p* = .003), and daytime dysfunction (*p* < .001) were found between the groups. Moreover, the significant differences identified between the two groups at the 2- and 4-week (*p* < .05), but not at the 8-week, postintervention time points demonstrate that, although the AA group had improved significantly over the SHI group in terms of global sleep quality and sleep latency during and at the end of the intervention period (i.e., 2 and 4 weeks), these improvements were not sustained at 8 weeks posttest. Similarly, improvements in subjective sleep quality and daytime dysfunction in the AA group began at 2 weeks and remained through 4 weeks. However, no significant differences were observed between the groups in terms of mean changes in the other outcome measures.

**Table 2.** Comparison of Outcome Variables at Baseline Between Groups (*N* = 179)

Variable	Acupressure Group (*n* = 88)		SHI Group (*n* = 91)		*t*	*p*
	Mean	*SD*	Mean	*SD*		
PSQI global score	11.91	3.34	11.24	3.41	1.32	.188
Sleep latency	2.58	0.88	2.29	0.89	1.45	.149
Habitual sleep efficiency	2.13	1.11	1.74	1.16	2.28	.024
Sleep disturbances	1.02	0.40	1.23	0.54	−2.93	.004
Subjective sleep quality	2.10	0.76	2.09	0.55	0.15	.885
Sleep duration	2.17	0.92	1.36	0.90	0.63	.528
Daytime dysfunction	1.27	0.92	1.36	0.90	−0.66	.509
Sleep medication	0.74	1.23	0.47	1.02	1.58	.116
Physical Component Summary-12	45.63	8.92	46.25	9.72	−0.44	.658
Mental Component Summary-12	43.27	8.12	43.36	7.83	−0.08	.939

*Note*. SHI = sleep hygiene instruction; PSQI = Pittsburgh Sleep Quality Index.

**Table 3.** Between-Group Comparison of the Effects of Acupressure and Sleep Hygiene Instructions on Outcome Measures (*N* = 179), Using Linear Mixed-Model Analysis

Measure	Acupressure Group (*n* = 88)		Mean Change	SHI Group (*n* = 91)		Mean Change	Difference in Mean Change Between Groups Over Time	*p*^a^
	Mean	*SE*		Mean	*SE*			
PSQI global score								.022
Baseline	11.91	0.36	—	11.24	0.35	—	—	
2-week posttest	8.98	0.40	−2.93	10.31	0.38	−0.93	−2.00**	
4-week posttest	8.25	0.43	−3.66	9.60	0.40	−1.64	−2.02*	
8-week follow-up	8.49	0.47	−3.42	8.83	0.42	−2.41	−1.01	
Sleep latency								.042
Baseline	2.48	0.94	—	2.29	0.09	—		
2-week posttest	1.94	0.11	−0.54	2.18	0.10	−0.11	−0.43*	
4-week posttest	1.86	0.10	−0.62	2.12	0.10	−0.17	−0.45*	
8-week follow-up	1.97	0.11	−0.51	1.88	0.10	−0.41	−0.10	
Habitual sleep efficiency								.936
Baseline	2.13	0.12	—	1.74	0.12	—		
2-week posttest	1.51	0.13	−0.62	1.24	0.12	−0.50	−0.12	
4-week posttest	1.39	0.14	−0.74	1.12	0.13	−0.62	−0.12	
8-week follow-up	1.46	0.14	−0.67	1.08	0.13	−0.66	−0.01	
Sleep disturbances								.378
Baseline	1.02	0.05	—	1.23	0.05	—		
2-week posttest	1.05	0.05	0.03	1.19	0.05	−0.04	0.07	
4-week posttest	1.00	0.05	−0.02	1.07	0.05	−0.16	−0.14	
8-week follow-up	1.05	0.05	0.03	1.11	0.04	−0.12	−0.09	
Subjective sleep quality								.003
Baseline	2.10	0.07	—	2.09	0.07	—		
2-week posttest	1.44	0.07	−0.66	1.91	0.07	−0.18	−0.48**	
4-week posttest	1.35	0.07	−0.75	1.74	0.07	−0.35	−0.40**	
8-week follow-up	1.34	0.07	−0.76	1.68	0.07	−0.41	−0.35*	
Sleep duration								.210
Baseline	2.17	0.11	—	2.08	0.10	—		
2-week posttest	1.61	0.12	−0.56	1.85	0.11	−0.23	−0.33	
4-week posttest	1.42	0.12	−0.75	1.78	0.12	−0.30	−0.45*	
8-week follow-up	1.42	0.12	−0.75	1.57	0.11	−0.51	−0.24	
Daytime dysfunction								< .001
Baseline	1.27	0.10	—	1.36	0.10	—		
2-week posttest	0.83	0.09	−0.44	1.46	0.09	0.10	−0.54**	
4-week posttest	0.52	0.09	−0.75	1.38	0.08	0.02	−0.77***	
8-week follow-up	0.54	0.09	−0.73	1.11	0.08	−0.25	−0.52**	
Sleep medication								.815
Baseline	0.74	0.12	—	0.47	0.12	—		
2-week posttest	0.60	0.12	−0.14	0.48	0.11	0.01	−0.13	
4-week posttest	0.71	0.12	−0.03	0.39	0.11	−0.08	0.05	
8-week follow-up	0.70	0.13	−0.04	0.41	0.11	−0.06	0.02	
PCS-12								.224
Baseline	45.63	0.95	—	46.25	0.98	—	—	
2-week posttest	47.64	0.92	2.01	46.18	0.86	−0.07	2.08	
4-week posttest	47.93	0.93	2.30	45.02	0.86	−1.23	3.53	
8-week follow-up	48.78	0.90	3.15	46.07	0.82	−0.18	3.33	
MCS-12								.176
Baseline	43.27	0.85	—	43.36	0.84	—	—	
2-week posttest	45.69	0.89	2.42	43.97	0.84	0.61	1.81	
4-week posttest	48.14	0.72	4.87	44.87	0.66	1.51	3.36*	
8-week follow-up	46.51	0.81	3.24	45.17	0.73	1.81	1.43	

*Note.* SHI = sleep hygiene instruction; PSQI = Pittsburgh Sleep Quality Index; PCS-12 = Physical Component Summary-12; MCS-12 = Mental Component Summary-12.

^a^*p* value from Group × Time interaction (auricular acupressure group vs. sleep hygiene instruction group).

*P* values of difference in mean change between groups over time. *p < .05. ** *p* < .01. *** *p* < .001.

**Figure 2.**
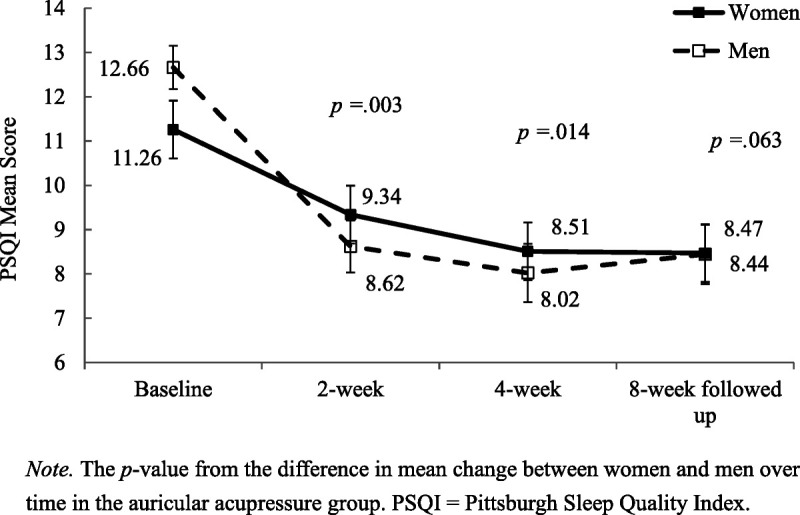
Mean scores on Pittsburgh Sleep Quality Index (PSQI) From Baseline to 8-week Posttest, with Standard Error for the Auricular Acupressure Group

The respective mean scores for global sleep quality for the women and men participants in the AA group from baseline through the 8-week follow-up are presented in Figure 2. The global score of PSQI decreased by 4.64 in the men participants and by 2.75 in the women participants in the AA group from baseline to 4 weeks posttest, with significant intergender differences at these two time points (*p* = .014) and no differences at 8 weeks posttest (*p* = .063).

## Discussion

The findings of this study demonstrate both the positive effects of a 4-week AA intervention on global sleep quality in community-dwelling adults older than 45 years of age who were poor sleepers and the lack of adverse effects in both the AA and SHI groups. These findings are similar to the results of a double-blinded, randomized controlled study conducted by Kao et al. (2012), which found that an AA intervention conducted 4 times per day over 4 weeks (shenmen and subcortex points) had a significant and positive effect on insomnia (as measured by reductions in oral doses of hypnotic drugs) but not on quality of life (as measured using the SF-36). The results of this study also partially echo those of Chueh et al. (2018), who used a single-group repeated measures design to examine the effects of a 4-week AA in a group of second-year RN-BSN women students (*n* = 36) and found that sleep quality began to improve significantly at the end of the second week of AA and continued through the remainder of the intervention. Furthermore, Chueh et al. found that depressed mood and anxiety improved significantly in each subsequent week of the intervention. This latter result is contrary to that of this study, which found no significant between- or within-group differences in perceived physical or mental health status. This discrepancy in findings may be related to the characteristics of the participants and the materials used in the AA intervention. In Chueh et al. study, the sample comprised second-year women RN-BSN students with a mean age of 32 years, suggesting that they may have already had experience with or knowledge of TCM techniques in hospital or university settings. Moreover, the magnetic pellet was taped and targeted with prolonged stimulation on the acupoint (Chueh et al., 2018; Lo et al., 2013). In contrast, the participants in this study had a mean age of 65.5 years and had a junior high school or lower level of education, and more than half were diagnosed with at least one chronic disease. In addition, those in the AA group had an embedded semen vaccaria seed on the ear acupoint and were instructed to press the acupoint 4 times per day. Although participants in the AA group were asked to make regular entries in their acupressure diaries, most did not due to age-related eye problems such as presbyopia and dry eye and/or to being illiterate or semiliterate. Thus, the researchers were able to confirm the accuracy and compliance of participants in the AA intervention only when the adhesive patches were replaced. Moreover, the intervention did not result in significant improvements in the physical and mental health-related chronic disease symptoms of the participants. Therefore, it is suggested that subjects who have no comorbidities be recruited in future studies to exclude potential confounders of health status. Furthermore, healthcare providers should develop strategies to assist older participants who self-administer AA at home.

Prolonged sleep latency, short sleep duration, and poor subjective sleep quality were the three most prevalent sleep problems reported by participants in this study. Notably, after 2 weeks of the AA intervention, significant effects were observed in terms of reduced sleep latency, improved subjective sleep quality, and improved daytime dysfunction. These effects were even more pronounced at 4 weeks posttest. This finding is similar to the results of previous studies of end-stage renal disease patients (Tsay & Chen, 2003) and older adult nursing home residents (Reza et al., 2010). Tsay and Chen (2003) implemented a 9-minute, 3 times per week acupoint massage intervention on the HT7 points on the ears and hands and on the Yungchuan points in both feet for 4 consecutive weeks, reporting significantly greater improvements in subjective sleep quality, sleep duration, habitual sleep efficiency, and sleep sufficiency in the intervention group than in the control group at 4 weeks postintervention. Also, Reza et al. (2010) evaluated an acupressure intervention that addressed four acupoints on the head, ears, and both of the hands and feet, reporting significant improvements in subjective sleep quality, sleep latency, sleep duration, habitual sleep efficiency, and sleep disturbance. Moreover, the findings of this study are supported by a prior systematic review and meta-analysis that evaluated the effects of acupressure on quality of sleep and determined that the greatest effects were achieved on sleep latency and sleep duration (Waits et al., 2018). In following up on the longer term (8 weeks posttest) effects of self-administered AA, significant differences were detected between the 8-week posttest values and the baseline values in terms of subjective sleep quality and daytime dysfunction. Few studies have explored the long-term effects of AA on sleep. Chen et al. (1999) used a randomized block experimental design to follow the 1-week effects in institutionalized older residents of an AA intervention that applied acupressure for 15 minutes per session, twice a day for 5 days per week over 3 consecutive weeks. The five acupoints used included baihui, fengchi, and anmian in the head and HT7 in the ears and hands. They collected data using the PSQI at pretest, at the end of the first week, and at posttest (Week 5), associating the intervention with significant improvements in the PSQI subscale scores of subjective sleep quality, sleep latency, sleep duration, habitual sleep efficiency, and global PSQI scores at posttest (Chen et al., 1999). To further enhance scholarly knowledge regarding the effectiveness of AA, additional studies should be conducted to compare the long-term effects of acupressure on HT7 ear points only with the long-term effects of acupressure on HT7 ear points, in combination with other acupressure points.

This was the first study to elucidate the effects of AA in improving global sleep quality among men and women who reported poor sleep. At the end of the second week of the 4-week AA intervention, the global sleep quality of the men participants had improved significantly more than that of the women participants. Furthermore, the most significant improvement was recorded 4 weeks after the intervention. It is well known that the prevalence of pain and chronic illness increases and gonadal hormone levels abate in postmenopausal women, leading to more severe insomnia for women in the same age range (Y. Liu et al., 2013; Lund & Lundeberg, 2008). In addition, women and men hold different expectations of various nonpharmacological interventions (Lund & Lundeberg, 2008). In this study, AA was found to have significantly different effects on women and men. Healthcare professionals should conduct qualitative studies to further explore the specific expectations of AA among middle-aged and older women and incorporate the results of this study into an AA protocol that is tailored to this important subgroup of care recipients.

One important limitation of this study was that the participants were not randomly assigned into the two groups due to the impracticality of blinding participants to group allocation because of the high likelihood of information sharing within each of the selected communities. Cluster randomization trials are recommended to test the effect of an intervention when individuals are naturally found in groups such as communities (Darlington & Donner, 2007). A second important limitation was the lack of an adequate control such as a sham group that receives a placebo intervention on nonspecific ear acupoints to rule out the effects of heightened expectations and the placebo effect. In this type of investigation, a sham group, as described, would be difficult to implement because of the large number of identified acupoints on the ear, which may confound the effects of the actual intervention (Zhang et al., 2014). Moreover, giving a sham intervention to a control group may raise ethical concerns in cancer patients, older adults, and other vulnerable populations (Azad & John, 2013; Cheng et al., 2015). Another limitation of this study was the use of self-reported data to measure sleep quality. However, subjective assessments of sleep problems may better reflect negative cognitive viewpoints or pessimistic thinking than objective measures and thus be suited for use with nonclinical samples (Grandner et al., 2006). In light of these limitations, study rigor was improved in several ways. First, a clustered and randomized controlled trial design was used to prevent experimental contamination. Second, those who administered the interventions in this study were not the investigators who collected data. Furthermore, the persons who administered the interventions and collected data, respectively, remained the same for each group in order to monitor participant compliance and reduce investigator bias. Third, the control group was provided with the standard SHI information sheet provided by healthcare organizations in Taiwan. Fourth, a linear mixed-model analysis was used to reduce the impact of missing data. Finally, a longitudinal observation was conducted to determine the onset and remaining effects on seven components of the PSQI and on perceived physical and mental health for the benefit of future scientific research into AA clinical practice.

### Conclusions

The results of this study indicate that a 4-week self-administered acupressure intervention on the ear HT7 points may be an effective and convenient intervention to improve the sleep quality of adult (> 45 years old) community dwellers with poor sleep, with improvements in subjective sleep quality and daytime dysfunction persisting for up to 4 weeks postintervention. Self-administered acupressure may be more effective in improving the sleep quality of men than women. For older adults and individuals with lower levels of formal education, single acupoint therapies represent an appropriate and easy-to-implement acupressure intervention that may be self-administered in community settings. Community healthcare professionals should work with CM physicians and other TCM resources in the community to identify appropriate acupoints and tailor AA self-management programs to the needs of each patient. As gender bias may influence research results and lead to inappropriate generalizations, future studies that are performed to build basic scientific evidence should include considerations of the effects of gender in the study design.
